# Cohort profile: Working age adults accessing secondary mental healthcare services in South London (UK) and benefits – A data linkage of electronic mental healthcare records and benefits data

**DOI:** 10.23889/ijpds.v8i2.2225

**Published:** 2023-09-14

**Authors:** Phillips Ava, Ray Leal, Sarah Dorrington, Matthew Hotopf, Matthew Broadbent, Amelia Jewell, Johnny Downs, Sharon Stevelink

**Affiliations:** 1 King's College London, London, United Kingdom; 2 NIHR Maudsley Biomedical Research Centre, London, United Kingdom

## Objectives

To present an overview of a cohort of working age adults accessing secondary mental healthcare services and benefits related to unemployment, sickness, disability, or income support and describe the different benefit types received across diagnostic and sociodemographic groups.

## Method

Using a novel data linkage containing electronic secondary mental health care records from the South London and Maudsley (SLaM) NHS Foundation Trust and benefits data from the Department for Work and Pensions (DWP), we present descriptive statistics on a cohort of working age adults. The data window covers the period January 2007-June 2020.

## Results

We identified n=150,348 patients of working age (18-65 years), who had attended SLaM secondary mental health care services, 78.3% of which had received a benefit relating to unemployment, sickness, disability, or income support. Of this group, 68% had a recorded primary psychiatric diagnosis. We found that a much higher percentage of those with a primary psychiatric diagnosis received more than one benefit (69.4%) compared to those who had not received a primary psychiatric diagnosis (30.6%).

## Conclusion

We showed types of benefits received among working age adults accessing secondary mental health care services. This cohort will be further examined to explore trajectories of mental health care and benefit receipt and provide evidence that will help to inform both DWP policies and mental health care delivery.

